# Oral Health Intervention Using Behavioral Change Techniques for Mild Dementia: A Mixed Method Study

**DOI:** 10.1093/geroni/igaf122.1025

**Published:** 2025-12-31

**Authors:** Sophie Zhijing Xu, Eunjung Ko, Jing Wang, Jackie Finik, Shahrzad Siamdoust, Justine Seidenfeld, Ya-Ning Chan

**Affiliations:** New York University, New York, New York, United States; New York University, New York, New York, United States; University of New Hampshire, Durham, New Hampshire, United States; New York University, New York, New York, United States; New York University, New York, New York, United States; Durham VA Healthcare System, Durham, North Carolina, United States; Duke University, Durham, North Carolina, United States

## Abstract

Maintaining oral health in individuals with mild dementia (PLwMD) is challenging due to cognitive impairments that hinder independent adherence to oral hygiene routines. To encourage PLwMDs’ engagement in oral hygiene self-care behaviors, implementing behavioral change techniques (BCTs) are essential. Despite the effectiveness of BCT-based interventions in various health contexts, their application in PLwMD has been underexplored. To address this gap, this study evaluated the effect of a care partner-assisted intervention designed to improve oral health outcomes through structured coaching sessions incorporating BCTs. Seventeen care partners–PLwMD dyads participated in four monthly coaching sessions led by an interventionist and a dental hygienist. A mixed-methods approach was employed, including 1) deductive content analysis of coaching session transcripts to identify BCT applications and 2) quantification of BCT occurrences based on code frequency. Additionally, gingival index changes from baseline to the 3-month follow-up were used to categorize dyads as ’no change (n = 3)’, ‘worsened (n = 2)’, and ‘improved (n = 12)’. Preliminary results showed that the most prevalent BCT codes were goal setting, problem-solving, and action planning, etc. This suggests that greater use of BCTs drives positive behavior change, reinforcing better oral hygiene practices and improving oral health outcomes. The finding also highlights the potential of BCT-based interventions to enhance self-care behaviors for oral health in PLwMD. Further exploration is warranted to optimize care partner training and intervention strategies to maximize and sustain improvements in oral health outcomes for PLwMD.

